# Association of an empty sella and grave´s disease in a patient with acromegaly: a case report

**DOI:** 10.11604/pamj.2021.38.394.25034

**Published:** 2021-04-22

**Authors:** Imen Halloul, Asma Ben Abdelkerim, Ghada Saad, Ahlem Slim, Yosra Hasni, Wafa Ben Othman, Maha Kacem, Molka Chaieb, Amel Maaroufi, Koussay Ach

**Affiliations:** 1 Endocrinology and Diabetes Department, Farhat Hached University Hospital, Sousse, Tunisia

**Keywords:** Acromegaly, empty sella, hyperthyroidism, grave´s disease, case report

## Abstract

Acromegaly is, in most cases, caused by growth hormone secreting pituitary adenomas. Those patients often develop different pathologies of the thyroid gland, however, the occurrence of Grave´s disease is quite a rare situation. We report a case of a 64-year-old female patient who presented with signs of hyperthyroidism and imbalance of her diabetes mellitus. On physical examination, she had facial features of acromegaly. Biochemical testing confirmed the suspicion of acromegaly and Grave´s disease, with an elevated insulin-like growth factor-1 and a suppressed thyroid stimulation hormone (TSH) with positive TSH-receptor antibodies. A pituitary Magnetic Resonance Imaging (MRI) was performed, revealing a macro-adenoma and an empty sella. The patient successfully underwent a transsphenoidal surgery and obtained a remission of her hyperthyroidism under anti-thyroid drugs.

## Introduction

Acromegaly is a systemic disorder caused by an excessive secretion of the growth hormone (GH). It is caused by a pituitary adenoma (PA) in most cases [1]. The association of empty sella (ES) and GH secreting pituitary adenoma has been described and multiple physiopathological theories have been suggested. Moreover, the thyroid gland is affected in patients with acromegaly, both in terms of endocrine function disturbances and structural alterations [2]. However, the prevalence of Grave´s disease (GD) in these patients is quite a rare situation [3]. Here we report an unusual case of a patient diagnosed with acromegaly secondary to a PA, which had an empty sella on magnetic resonance imaging (MRI) and presented with hyperthyroidism due to GD.

## Patient and observation

**Figure 1:**
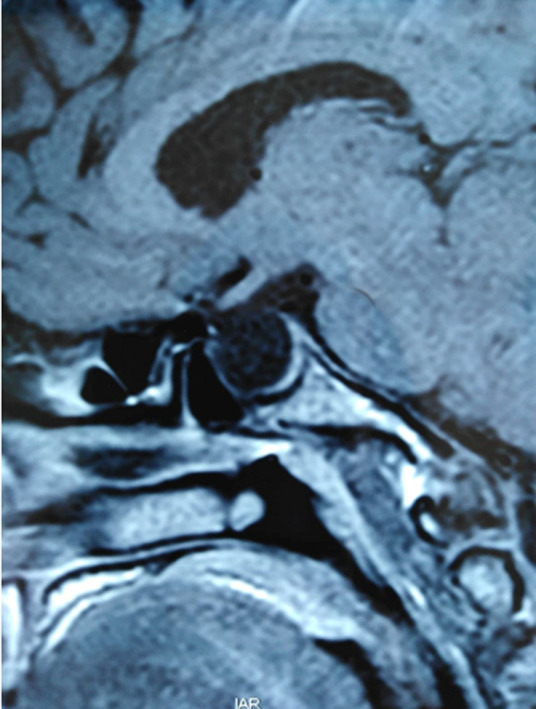
sagittal spin echo T1-weighted image showing the macro-adenoma

A 64-year-old female patient was referred to the endocrinology department for investigation of recent diabetes mellitus imbalance and symptoms of hyperthyroidism. For her personal medical history, she has been treated for hypertension and sleep apnea for 17 years now, and was operated on gonarthrosis and carpal tunnel syndrome in both hands 15 years ago. Regarding her family history, her first daughter was diagnosed with GD and the second daughter was followed for hyperprolactinemia caused by an empty pituitary sella. Clinically, she was complaining of thermophobia, trembling, polyuro-polydipsic syndrome and weight loss. On examination, she had facial features of acromegaly with enlargement of her hands and feet and a goiter. Her blood pressure was often high. She did not present with specific skin lesions or bones deformations. Biochemical testing revealed an elevated insulin-like growth factor-1 (IGF1) and a suppressed thyroid stimulation hormone (TSH) with positive TSH-receptor (TSHR) antibodies. The prolactin level was normal and corticotropic insufficiency was ruled out with a normal level of cortisol after 1μg Synacthen test (Table 1). A pituitary MRI was performed, revealing a 12-mm adenoma with ES and no signs of local invasion (Figure 1). No history of pituitary apoplexy was described by the patient. A transsphenoidal surgery was successfully performed and the diagnosis of GH-producing adenoma was also confirmed with immune-histochemistry. For her hyperthyroidism, she was treated with anti-thyroid drugs with a fast remission after 4 months of treatment.

**Table 1:** hormones work-out results of the patient

Hormones level	Values
IGF1 (ng/ml)	800
Prolactin (ng/ml)	13.9
FSH (mUI/ml)	41
LH (mUI/ml)	21.7
Estradiol (pg/ml)	< 10
TSH (μUI/ml)	< 0.05
FT4 (pmol/l)	32.30
PTH (pg/ml)	28.5
Basal Cortisol (ng/ml)	118.4
Cortisol after adrenocorticotropic hormone stimulation (ng/ml)	315

TSH: Thyroid-stimulating hormone, FSH: Follicle Stimulating Hormone, LH: luteinizing hormone, IGF1: insulin like growth factor 1, PTH: parathyroid hormone, FT4: Free Thyroxine 4

## Discussion

Association between ES and acromegaly was described for the first time in the 1960s [4]. Many reports came after to elaborate the mechanisms of this situation. In fact, ES results from the herniation of the subarachnoid space within the sella turcica, which is often associated with some degree of flattening of the pituitary gland [5]. Primary ES has to be distinguished from secondary ES, which is caused by iatrogenic factors such as surgery, radiation or medical treatment [6]. Liu *et al*. found in his study that GH-producing microadenomas with ES account for 20% of all growth hormone microadenoma cases [7]. Mainly 3 physiological theories have been proposed to explain this association. The first hypothesis would be a pituitary gland or tumor apoplexy [8]. Secondly, Bier *et al*. suggested that the growth pattern of GH-secreting pituitary tumors and accompanying sellar floor remodeling may induce the morphological ES aspect in patients with acromegaly [9]. This theory was actually inspired by the fact that sellar enlargement could be the result of a paracrine role of local GH on bones. Possibly, the local sellar bone changes (paracrine GH action) may be distinct from the general bone changes (systemic/endocrine GH and IGF-1 action) observed in patients with acromegaly. The systemic changes are characterized by increased bone turnover (bone formation and bone resorption, usually resulting in increased appositional bone growth and cortical thickening) and mediated by endocrine and locally produced IGF [10]. Furthermore, this proposition was supported by the publication of Mnif Fekih *et al*. about an ES associated with an ectopic secretion of growth hormone releasing hormone by a pancreatic neuroendocrine tumor [11]. Finally, ES may be caused in acromegalic patients by the intra-sellar herniation of the supra-sellar subarachnoid spaces and by the sellar enlargement due to infra-sellar extension of GH-secreting adenomas [9, 12, 13].

As for our patient, she did not present with any history of pituitary apoplexy nor did she suffer from any hormonal hyposecretion. Therefore, the second theory would probably be the case. Sasagawa *et al*. studied the impact of ES on transsphenoidal surgery in such patients and he concluded that they might encounter a higher risk for non curative resection in surgical treatment and they may also present with more frequent intra-operative cerebrospinal fluid leakage [12]. Our patient also presents an unusual association with GD. In fact, the endocrine function and the structure of the thyroid gland are affected in patients with acromegaly. The rate of non-autoimmune forms of hyperthyroidism in acromegaly ranges between 5 and 14.7%, and up to 20% within the subgroup of patients with a thyroid nodular goiter [14]. As for the GD, it is only present in 1% of the cases. Di Cerbo *et al*. studied the role GH and IGF1 play in influencing the production of auto-antibodies directed against the TSH receptor (TSHR), therefore, exacerbating TSHR-induced Graves´ thyrotoxicosis [3]. As a matter of fact, GH and IGF1 potentiate the actions of TSHR antibodies by 3 mechanisms. Firstly, they stimulate T cell proliferation and inflammatory cytokine production by T cells infiltrating the thyroid [15]. Secondly, they promote B cell immunoglobulin production and proliferation and finally, they incite post receptor pathways directly in thyroid cells [3].

## Conclusion

In conclusion, the present case illustrates the uncommon occurrence of acromegaly, empty sella and Grave´s disease. Hyperthyroidism in our patient was probably due to an auto-immune background with the multiple effects of GH and IGF1 on the thyroid and the immune system. As for the ES, it has been described in multiple studies and they all concluded to tumor-induced local bone remodeling processes or an episode of apoplexy.
